# Motivation to Clinical Trial Participation: Health Information Distrust and Healthcare Access as Explanatory Variables and Gender as Moderator

**DOI:** 10.3390/jcm14020485

**Published:** 2025-01-14

**Authors:** Rifath Ara Alam Barsha, Shervin Assari, Samuel Byiringiro, Erin D. Michos, Timothy B. Plante, Hailey N. Miller, Cheryl R. Himmelfarb, Payam Sheikhattari

**Affiliations:** 1 Division of Cardiology, Johns Hopkins University School of Medicine, Baltimore, MD 21205, USA; rbarsha1@jh.edu (R.A.A.B.); edonnell@jhmi.edu (E.D.M.); 2 Department of Internal Medicine, Charles R. Drew University of Medicine and Science, Los Angeles, CA 90059, USA; shervinassari@cdrewu.edu; 3 Department of Urban Public Health, Charles R. Drew University of Medicine and Science, Los Angeles, CA 90059, USA; 4 Marginalization-Related Diminished Returns Center, Los Angeles, CA 90059, USA; 5 Johns Hopkins University School of Nursing, Baltimore, MD 21205, USA; sbyirin1@jhu.edu (S.B.); hmille45@jhu.edu (H.N.M.); chimmelfarb@jhu.edu (C.R.H.); 6 Department of Medicine, Larner College of Medicine at the University of Vermont, Burlington, VT 05405, USA; timothy.plante@uvm.edu; 7 Department of Health, Behavior and Society, Johns Hopkins University Bloomberg School of Public Health, Baltimore, MD 21205, USA; 8 School of Community Health and Policy, Morgan State University, Baltimore, MD 21251, USA

**Keywords:** clinical trial participation, underrepresented population, diversity, motivation, gender differences, health system

## Abstract

**Background**: There is significant underrepresentation in clinical trials across diverse populations. Less is known about how health system-related factors, such as relationships and trust, mediate the motivation for clinical trial participation. We aimed to investigate whether health system-related factors explain the association between sociodemographic factors and motivation for participation. Additionally, we explored whether the mediating effects differ by gender. **Methods**: We used the Health Information National Trends Survey 2020 cycle-4 data. Motivation for clinical trial participation, assessed through eight items, was the outcome variable (range 1–4). Predictors included age, race, ethnicity, education, general health, and depression. The health system-related explanatory variables were health information distrust, having a regular provider, and the frequency of healthcare visits. Gender was the moderator. A structural equation model (SEM) was used for the overall and gender-stratified analyses. **Results**: Among the 3865 participants (mean [SE] age of 48.4 [0.53] years, 51.4% women, and 24.3% non-White), older age (β = −0.170; *p* < 0.001) and non-White race (β = −0.078; *p* < 0.01) were negatively associated, and higher education (β = 0.117; *p* < 0.001) was positively associated with motivation. Higher distrust (β = −0.094; *p* < 0.01) decreased motivation, whereas having a regular provider increased motivation (β = 0.087; *p* < 0.01). The gender-stratified SEM revealed that women, but not men, with higher distrust showed lower motivation (β = −0.121; *p* < 0.01), and men, but not women, with a regular healthcare provider showed higher motivation (β = 0.116; *p* < 0.01). **Conclusions**: Our study found that women with higher distrust showed lower motivation, while men with a regular healthcare provider demonstrated higher motivation. These gender differences highlight the need for tailored recruitment approaches that account for their distinct relationships with the health system.

## 1. Background

### 1.1. Clinical Trial Diversity

Clinical trial diversity is essential while testing novel treatments to ensure safety and efficacy across diverse populations. However, in the United States (US), several populations are excluded or underrepresented in clinical trials, including women and racial and ethnic minority populations. Women of reproductive age are often systematically excluded from many studies [1,2]. Excluding pregnant and lactating women remains a common practice in clinical trials [1]. In addition, there is a significant underrepresentation of racial and ethnic minority populations, with the predominant recruitment of White, socioeconomically advanced participants in clinical trials [3,4].

Research has shown that the underrepresented groups in clinical trials may have distinct health circumstances that interfere with their response to new drugs or interventions, undermining the findings’ generalizability and the safety and efficacy of such treatments [5]. For example, as a treatment of depression, tricyclic antidepressants were found to be more effective in men, while women responded more to selective serotonin reuptake inhibitors [6,7]. Thus, improving the diversity in clinical trials is critical to ensure the safety and efficacy of novel treatments.

### 1.2. Current Efforts to Promote Diversity and Persistent Underrepresentation

Historically, clinical trials often excluded participants from populations most affected by specific conditions, diseases, or behaviors [8]. Instead, these trials predominantly recruited White male participants, resulting in significant gaps in understanding the disease pattern, prevention, and treatment effectiveness of conditions and diseases across diverse populations [8]. However, increasing the diversity in clinical trials has been a policy priority for federal agencies over the past three decades.

Significant efforts have been made to improve the diversity of trial participants. For instance, the National Institutes of Health (NIH) and the US Food and Drug Administration (FDA) have implemented policies and provided guidelines to encourage and ensure the inclusion of diverse participants in clinical trials [9,10]. These initiatives have provided a strong foundation to promote diverse participation, yet many groups continue to be underrepresented. In 2020, among the 53 drug trials that led to FDA approval, only 8% of the participants were Black Americans, 11% were Hispanic individuals, and 30% were those aged 65 and older [11]. These figures have declined since 2019, when 9% of participants in FDA-approved drug trials were Black Americans, 18% were Hispanic, and 36% were older adults (aged 65 and older) [11], emphasizing the ongoing need for targeted efforts and strategies to increase diversity. 

Further, trends in women’s enrollment in clinical trials indicate progress in gender representation. Between 2014 and 2021, women accounted for an average of 51% of trial participants, with representation ranging from 37% in 2014 to 57.1% among drugs that received FDA approval [10]. Similarly, an NIH report highlighted that the average representation of women in clinical trials increased from 44.3% in 2013 to 52.4% in 2018 across all NIH institutes [10]. Despite this increasing representation, women remain underrepresented in certain types of clinical trials. Data on women’s participation in clinical trials by trial type revealed that women have continued to be underrepresented in clinical trials for cancer, chronic kidney disease, and most cardiovascular conditions [12,13]. These highlight the significant gaps in clinical trial diversity despite the ongoing efforts and policies. Addressing these gaps requires examining the motivational factors that influence participation.

### 1.3. Motivational Factors and Barriers

Motivation, which differs for each person, plays an important role in encouraging individuals to participate in clinical trials. Personal health benefits, access to new treatments, financial compensation, doctor recommendations, helping others, and the desire to contribute to science are among the various motivations identified in previous studies [14,15,16,17]. On the other hand, mistrust of the health system, lack of access to healthcare, and insurance barriers are some of the most commonly identified barriers [18,19]. These factors may vary considerably across different demographic subgroups. For example, Black communities have fostered a deep-seated mistrust of medical systems following historical events such as the Tuskegee Syphilis Study [20]. This mistrust continues to contribute to skepticism regarding research, including clinical trials. On the other hand, Black and Hispanic individuals are more likely to be uninsured compared to their White counterparts [21], limiting their access to healthcare and opportunities for participation in clinical trials.

In addition, cultural values and beliefs may significantly influence attitudes toward clinical trials. Language barriers can impact an individual’s ability to fully understand the benefits of clinical trials or trial protocols, posing a major obstacle [22] and further exacerbating mistrust in the research process. Moreover, cultural stigmas related to illness, particularly sexually transmitted diseases and mental health-related conditions, may negatively influence individuals’ perception about participation in research [22]. Socioeconomic status (SES) also influences participation in clinical trials. Individuals with lower SES often have financial barriers, such as the inability to take paid time off from work or lack of childcare, which can impact their ability and willingness to participate [23,24]. Moreover, individuals from lower SES groups may lack health insurance, further restricting their access to healthcare and participation in clinical trials [24]. In addition, there is growing use of digital tools and technologies for research and healthcare delivery, such as digital consent forms, telemedicine, and wearable devices, which presents additional challenges [25]. Individuals from lower SES groups often lack digital literacy or access to necessary devices or tools to effectively navigate these systems [25]. This “digital divide” may further exclude already underrepresented populations, thereby increasing the disparities.

Participants’ experiences and perceptions, as documented in previous studies, offer valuable insights into their views on clinical trials. One participant shared the following: “You are giving back. Even though this may not help you, it will help others in the future who have your disease or other diseases like diabetes, heart disease, cancer. This may help your children or your grandchildren. You can help save lives” [26]. In contrast, one participant documented mistrust as a significant barrier, stating: “All people are needed for studies to improve treatments and find cures. Targeting African American recruitment makes it appear suspicious” [26]. Insurance issues also emerged as a barrier, as noted by one respondent: “I had a couple of patients that their insurance wouldn’t cover clinical trials” [27]. 

Though research has shown that various sociodemographic and health factors, such as age, gender, race, ethnicity, education, and health status, significantly influence an individual’s willingness to participate in clinical trials [28,29], the role of health system-related factors, such as distrust in health information, having a regular healthcare provider, and the frequency of healthcare visits, as potential explanatory variables in this process have been less explored. The effects of these factors are not likely to be direct but operate through specific mechanisms. One such mechanism could be the relationship that individuals have with the healthcare system and the trust that they place in it. For instance, having a regular healthcare provider may foster trust and familiarity [30], which can indirectly influence an individual’s willingness to participate in clinical trials. By examining these mediating pathways, this study aimed to investigate the connection between sociodemographic and health factors and clinical trial participation. Addressing these gaps is a crucial step toward improving the diversity of trial participants.

### 1.4. Study Objectives

Therefore, this study investigated whether health information distrust, having a regular provider, and the frequency of healthcare visits mediate the association between sociodemographic and health factors and motivation to participate in clinical trials. Given the differences in participation rate among men and women, we also sought to test whether these explanatory variables operate differently for men and women, thereby offering more nuanced insights into the motivators of clinical trial participation.

Understanding these dynamics is critical for enhancing representation in clinical trials as well as for ensuring that the findings benefit all parts of society. This study aimed to address these gaps and provide actionable insights to guide policies and programs. This insight is crucial for developing targeted recruitment strategies and directing efforts toward specific subgroups of populations, ultimately contributing to more diverse and representative clinical trials.

## 2. Methods

### 2.1. Data Source

We used the Health Information National Trends Survey (HINTS) 2020 cycle 4 data [31]. HINTS is an ongoing, nationally representative survey conducted by the National Cancer Institute among US adults aged ≥18 years. HINTS utilizes a multistage, stratified sampling design to obtain information on health knowledge, attitudes, and behaviors, including some sociodemographic data [31]. The sampling strategy followed a two-stage design. In the first stage, a stratified sample of residential addresses was drawn from an address database. In the second stage, one adult was randomly selected from each sampled household by utilizing the Next-Birthday Method [31]. The sampling frame of addresses was divided into the following two distinct strata: (1) areas with a high concentration of minority populations and (2) areas with a low concentration of minority populations [31]. The HINTS 2020 Cycle 4 data were collected via mail between 24 February 2020 and 15 June 2020 using a survey instrument available in both English and Spanish. Each household received one English survey per mailing. If a member of the household contacted Westat and request a Spanish version of the survey, one Spanish survey was sent to that household per mailing for all future mailings. Participants were offered a USD 2 incentive (pre-paid) to encourage participation. Although the response rate of the HINTS study has been low (36.6% for this year) [31], it has been the source of knowledge for health services and the source of medical and health information among US adults [31]. Random selection of the participants was performed to ensure representation from diverse sociodemographic groups.

### 2.2. Ethical Considerations

The Westat Institutional Review Board (IRB) approved the original HINTS study (Project #6048.14). HINTS 4 was determined to be “Not Human Subjects Research” by the NIH Office of Human Subjects Research Protection (Exempt #13204) [32]. HINTS data are deidentified and publicly available. Therefore, our study was exempt from IRB review.

### 2.3. Study Sample

Our study sample included HINTS participants, comprising a total of 3865 adults aged 18 years and older.

### 2.4. Study Measures

#### 2.4.1. Outcome Variable

Motivation to Participate in Clinical Trials: The study’s outcome variable is the motivation to participate in clinical trials. HINTS asked its participants the following question: “Imagine that you had a health issue, and you were invited to participate in a clinical trial for that issue. How much would each of the following influence your decision to participate in the clinical trial?” The statements included 8 items and each item was assessed through a 4-point Likert scale (Not at all, A little, Somewhat, and A lot): (a) I would be helping other people by participating; (b) I would get paid to participate; (c) I would get support to participate such as transportation, childcare, or paid time off from work; (d) If my doctor encouraged me to participate; (e) If my family and friends encouraged me to participate; (f) I would want to get better; (g) I would get the chance to try a new kind of care; (h) If the standard care was not covered by my insurance. For each item, we coded the response options as 1 = Not at all, 2 = A little, 3 = Somewhat, and 4 = A lot. We then calculated the mean score of the eight items mentioned above. The mean score ranged from 1 to 4. A higher score indicated higher motivation. By aggregating the responses into a single mean score, we captured the overall motivation. We used this mean score as a continuous measure in the analysis with structural equation models (SEMs). We used the outcome as a continuous measure, a commonly preferred approach in SEMs, which work best with correlations and covariances rather than dichotomous outcomes [33]. However, for the descriptive analysis, the variable was used as a dichotomous measure (dichotomized at the median to create the binary variable: 0 = low motivation and 1 = high motivation). The median value is commonly used as a cut-off to dichotomize and generate two comparable groups [34,35]. This dichotomous categorization allowed us to compare high-motivated and low-motivated groups at baseline, providing valuable context for understanding the differences between these groups before conducting the SEM analysis. The Cronbach’s alpha of these 8 items was 0.84.

#### 2.4.2. Predictors

##### Sociodemographic Variables

Age, gender, race, ethnicity, and education were the sociodemographic variables. We used age in years as a continuous variable. Self-reported gender was used as a dichotomous measure (0 = Men and 1 = Women). We categorized race (self-reported) into the following two groups: White and non-White. This categorization was made to compare racial minorities with White individuals, as racial minorities often experience similar disparities when compared to White individuals [36,37,38]. Additionally, the sample size was too small for specific racial categories, such as Asian Americans, American Indians or Alaska Natives, Pacific Islanders, and individuals identifying with multiple races, to be included as distinct groups in the SEM path analysis. The non-White group included Black Americans, Asian Americans, American Indians or Alaska Natives, Pacific Islanders, and individuals of multiple races, and was coded as 1, with White serving as the reference category, coded as 0. We used this variable as a dichotomous measure in the SEM. While ethnicity was used as a binary measure (0 = non-Hispanic and 1 = Hispanic), we used educational attainment as an ordinal variable, ranging from 1 to 4 (1 = Less than high school, 2 = High school graduate, 3 = Some college, and 4 = College graduate or more). 

##### Health Status Variables

Depression and general health were the health status variables. The Patient Health Questionnaire-4 (PHQ-4), a four-item self-administered tool developed to assess depressive symptoms, was used to measure the depressive symptoms [39]. The PHQ-4 was tested in previous studies and found to be a valid and reliable measure of depression in the general US population. Further, the PHQ-4 has demonstrated strong psychometric properties, including high internal consistency and sensitivity, making it an appropriate and reliable measure for a diverse sample like the one in this study [40]. It has an internal consistency of 0.92 [41]. In addition, its use in various settings supports its applicability in understanding how depression may influence motivation [42,43]. Each item of the PHQ-4 was rated based on the frequency of symptoms experienced over the past two weeks, and the response options included: not at all (0), several days (1), more than half the days (2), and nearly every day (3). The sum of these four items was calculated, and the resulted total score ranged from 0 to 12. The summary score was used as a continuous measure, with higher scores indicating more severe depressive symptoms. General health status was assessed using the following question: “In general, would you say your health is—(1) excellent, (2) very good, (3) good, (4) fair, or (5) poor”. Those that responded ‘excellent’, ‘very good’, or ‘good’ were categorized as healthy and coded as 1. Those whose response was fair or poor were regarded as not healthy and coded as 0. This variable was used as the dichotomous measure in the analysis.

#### 2.4.3. Healthcare Relations Explanatory Variables

Health information distrust, having a regular provider, the frequency of healthcare visits, confidence in obtaining health information, quality of care, and communication with health professionals were the healthcare relations explanatory variables (Table 1).

### 2.5. Statistical Analyses

We followed the HINTS analytical guidelines for statistical analysis. The Taylor Series linearization method was used, provided by HINTS, to account for complex survey designs and generate nationally representative estimates of statistical measures [31]. We conducted univariate and bivariate analysis, and presented the results as weighted proportions and the mean with the standard error (SE) and a 95% confidence interval (CI). We used the SEM to perform the analysis in the pooled sample and stratified by gender with and without explanatory variables. We began our analysis by including health information distrust, having a regular provider, the frequency of healthcare visits, confidence in obtaining health information, quality of care, and communication with healthcare professionals as explanatory variables. However, confidence in obtaining health information, quality of care, and communication with healthcare professionals did not show significant results and did not contribute meaningfully to the models. In contrast, health information distrust, having a regular provider, and the frequency of healthcare visits remained significant explanatory variables. Therefore, we excluded the sets of non-explanatory variables (confidence in obtaining health information, quality of care, and communication with healthcare professionals) from the models to maintain the clarity and focus on the variables with meaningful contributions. The final models only included health information distrust, having a regular provider, and the frequency of healthcare visits as explanatory variables because they demonstrated clear and statistically significant associations with motivation. The results of the model, including all of the variables, are presented in Appendix A Table A1. The SEM was chosen for this study because it is a powerful multivariate technique and is a preferred method for testing indirect effects [44]. It allows for the simultaneous examination of multiple relationships. Additionally, the SEM is useful for comparing groups by conducting multi-group analyses to see if the same effects hold across different populations [44]. These capabilities make the SEM an ideal choice for capturing the complexity of the relationships examined in this study. The results of the SEM were presented as the beta coefficient (β), SE, and *p* values. We set the significance level at *p* ≤ 0.05. The goodness of fit of the model based on the measures of the residuals was estimated, and we reported the coefficient of determination for each model (Appendix A Table A2). The coefficient of determination is a widely used measure of the goodness of fit for linear models [45]. We did not exclude the missing data and conducted an available case analysis. In the SEMs, we addressed missing data using Full Information Maximum Likelihood (FIML) estimation. FIML is a robust method that utilizes all available data points to estimate model parameters, without requiring the imputation or deletion of cases with missing values [46]. Each path in the model used the maximum available data. Therefore, if an explanatory variable was missing in some cases, that missingness did not affect the estimation of other paths that did not include that variable. FIML is a reliable approach for managing substantial amounts of missing data while preserving statistical power and minimizing the risk of bias [46]. While the SEM is a powerful tool for examining complex relationships, it has its limitations. The SEM identifies associations and potential pathways but cannot establish causation. Stata 15.0 (StataCorp, LLC, College Station, TX, USA: StataCorp LLC) was used for the data analysis.

## 3. Results

### 3.1. Descriptive Statistics

Table 2 presents the descriptive results of the study in the overall sample and by motivation status.

The study sample included a balanced mix of men and women, with most of the participants identifying as White. Those with a high motivation to participate in clinical trials were generally younger and more likely to have a regular healthcare provider compared to those with low motivation. High-motivation participants also expressed greater confidence in obtaining health information and had more frequent healthcare visits. Despite these differences, both groups demonstrated similar levels of distrust toward health information.

The study sample (*n* = 3865; weighted *n* = 253,815,197) had a mean (SE) age of 48.4 (0.53) years, 51.4% were women, and 24.3% were of non-White race. A majority of the participants with high motivation were women (52.3%), while the percentage was the same for participants with low motivation. The mean (SE) age of the participants with low and high motivation were 50.1 (0.89) and 46.4 (0.73) years, respectively. The percentage of non-White participants was almost similar for those with low and high motivation, at 25.8% and 22.3%, respectively. While 4.2% of the participants with high motivation reported having less than a high school education, about 11.7% of the participants with low motivation reported similarly. About 66.1% of the participants with high motivation reported having a regular provider compared to 58.6% of the participants with low motivation. The percentage of participants not visiting a doctor in the past 12 months at least once was also higher among those participants with low motivation compared to those with high motivation (20.8% vs. 13.0%). The reporting of the quality of care was almost similar for both groups. While 38.4% of the participants with high motivation were very confident about obtaining health information, 32.0% of participants with low motivation reported similarly. The mean (SE) of health information distrust was the same for both groups, at 2.2 (0.02).

The observed differences between the high- and low-motivation groups highlight the key factors that may have influence on clinical trial participation. Participants with high motivation were more likely to have a regular healthcare provider, visit a doctor more frequently, and report higher confidence in obtaining health information. These findings suggest that access to healthcare and trust in health-related interactions play critical roles in fostering the motivation to participate.

### 3.2. Pooled SEM Results

**Figure 1:**
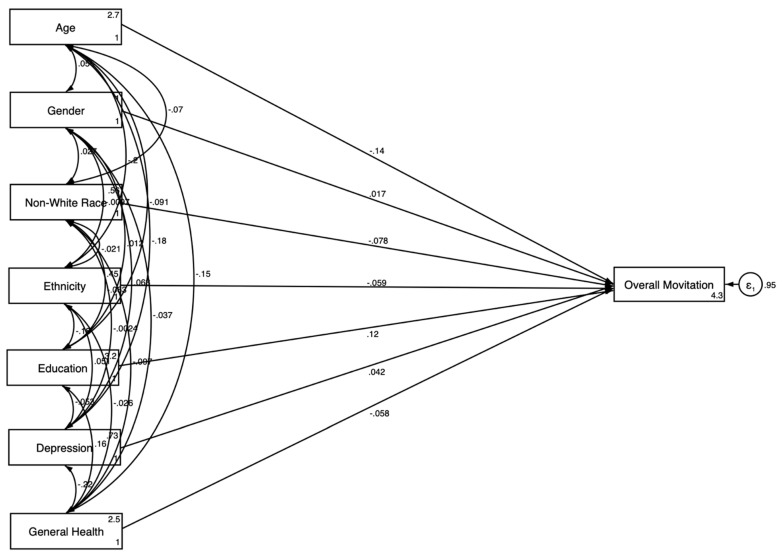
SEM results in the overall sample without explanatory variables.

**Figure 2:**
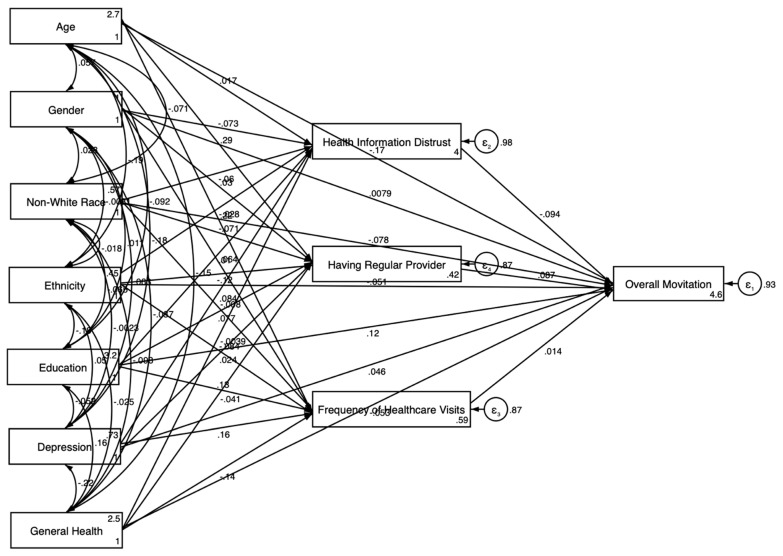
SEM results in the overall sample with explanatory variables.

While the descriptive statistics highlighted the key differences in motivation levels based on demographic and healthcare-related factors, we conducted SEM analyses to further explore these relationships and to understand how these factors influence the motivation to participate in clinical trials. The results of the SEM in the pooled sample are presented in Table 3 and Figure 1 and Figure 2.

The SEM results in the pooled sample revealed that older age and non-White race were significantly associated with the lower motivation to participate in clinical trials, while higher educational attainment was associated with a higher motivation. When health system-related factors were included in the model, these associations remained consistent. Additionally, good general health and higher distrust in health information were linked to lower motivation, whereas having a regular healthcare provider was associated with higher motivation. 

Model 1 is estimated in the pooled sample without the explanatory variables (Figure 1). The results showed that older age (β = −0.144; *p* < 0.001) and non-White race (β = −0.078; *p* < 0.01) were significantly negatively associated with the motivation to participate in clinical trials. Higher educational attainment was positively associated with motivation (β = 0.125; *p* < 0.001).

Model 2, which was estimated in the pooled sample with health relations explanatory variables (health information distrust, having a regular provider, and the frequency of healthcare visits), also found older age (β = −0.170; *p* < 0.001) and non-White race (β = −0.078; *p* < 0.01) to be negatively associated, and higher education (β = 0.117; *p* < 0.001) to be positively associated, with motivation. Good general health was negatively associated with motivation (β = −0.053; *p* < 0.05). Higher health information distrust (β = −0.094; *p* < 0.01) was negatively associated with motivation, while having a regular provider was positively associated (β = 0.087; *p* < 0.01). No significant association was found between the frequency of healthcare visits and motivation (Figure 2).

Figure 1 presents the results of the SEM without health relations explanatory variables, depicting the direct relationships between sociodemographic and health status factors and the overall motivation to participate in clinical trials. The model showed that older age and non-White race were negatively associated with motivation, while higher educational attainment was positively associated. Figure 2 expands on this by incorporating health system-related explanatory variables into the model. This model revealed both direct and indirect pathways, showing that having a regular healthcare provider was positively associated with motivation, whereas health information distrust was negatively associated.

### 3.3. Gender-Stratified SEM Results

**Figure 3:**
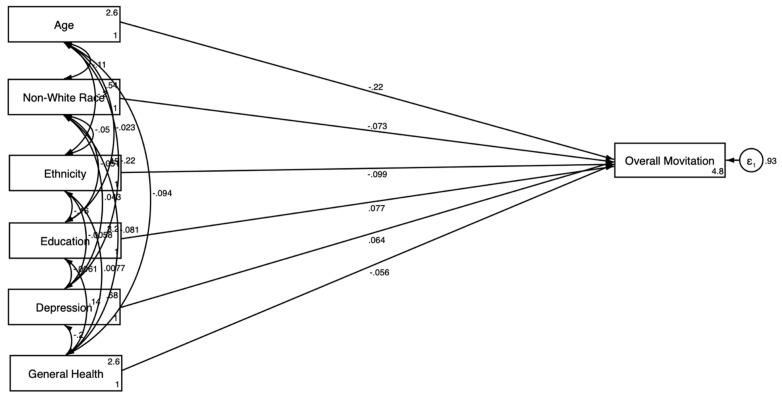
SEM results stratified by gender without explanatory variables—male.

**Figure 4:**
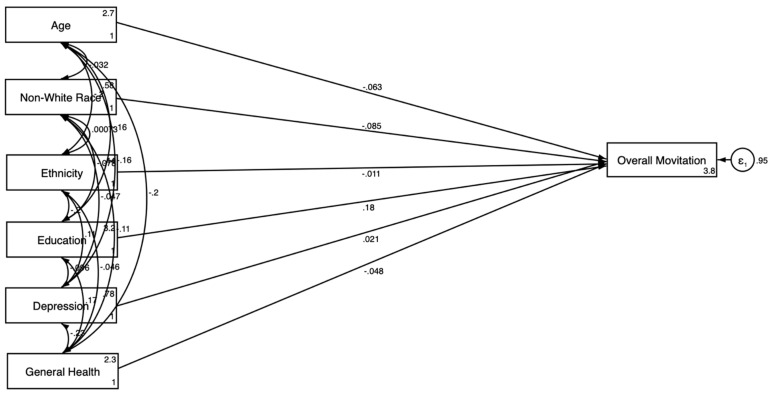
SEM results stratified by gender without explanatory variables—female.

**Figure 5:**
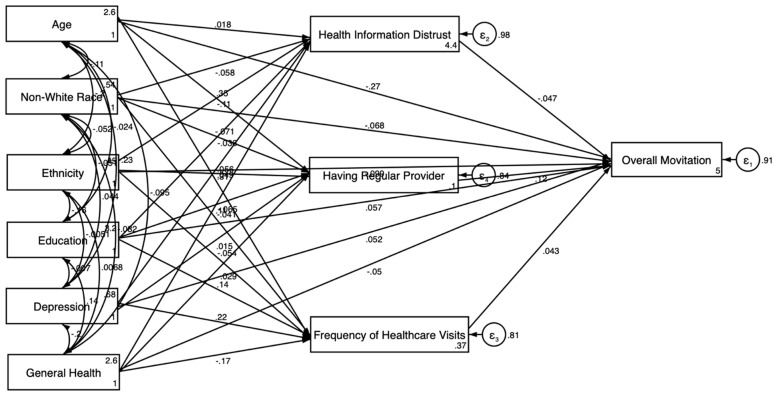
SEM results stratified by gender with explanatory variables—male.

**Figure 6:**
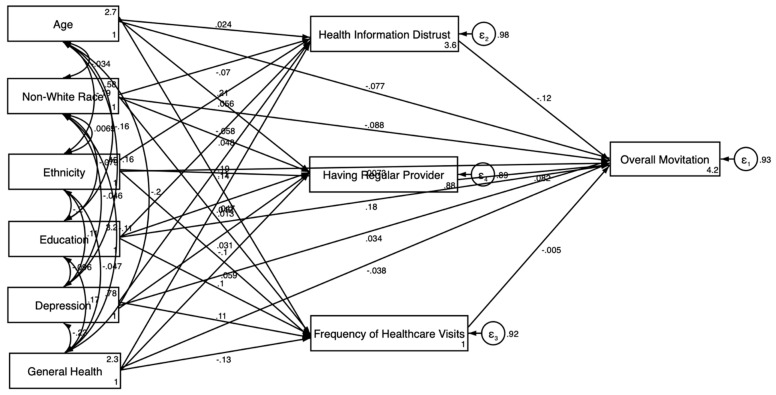
SEM results stratified by gender with explanatory variables—female.

Table 4 and Figure 3, Figure 4, Figure 5 and Figure 6 present the SEM results by gender.

The gender-stratified SEM results revealed distinct patterns for men and women. For men, older age, non-White race, and Hispanic ethnicity were negatively associated with the motivation to participate in clinical trials. When health system-related factors were included, older age and Hispanic ethnicity remained negatively associated, while having a regular healthcare provider showed a positive association with motivation. For women, higher educational attainment was positively associated with motivation, while non-White race was negatively associated. When health system-related factors were included, these associations remained, and higher distrust in health information was also linked to lower motivation.

#### 3.3.1. For Men

Model 3 is estimated without the explanatory variables and showed that older age (β = −0.219; *p* < 0.001), non-White race (β = −0.073; *p* < 0.05), and Hispanic ethnicity (β = −0.099; *p* < 0.05) were negatively associated with the motivation to participate in clinical trials for men (Figure 3). Model 4 was estimated with health relations explanatory variables. Older age (β = −0.275; *p* < 0.001) and Hispanic ethnicity (β = −0.099; *p* < 0.05) also showed a significant negative association with motivation in model 4 (Figure 5). Having a regular provider showed a significant positive association with motivation (β = 0.116; *p* < 0.01).

The SEM results for men are shown in Figure 3 and Figure 5. Figure 3 illustrates that older age and non-White race were negatively associated with motivation, while education had a positive association. Figure 5 incorporated explanatory variables, revealing both direct and indirect pathways. This model showed that health information distrust negatively influenced motivation, whereas having a regular healthcare provider positively influenced it.

#### 3.3.2. For Women

Model 5 is estimated without the explanatory variables. While higher education showed a significant positive association (β = 0.175; *p* < 0.001), non-White race (β = −0.085; *p* < 0.05) had a significant negative association with motivation for women (Figure 4). Model 6 included the health relations explanatory variables and showed that higher education was significantly positively associated (β = 0.178; *p* < 0.001) and non-White race (β = −0.087; *p* < 0.05) was significantly negatively associated with motivation (Figure 6). Health information distrust showed a significant negative association with motivation (β = −0.125; *p* < 0.01).

The SEM results for women are shown in Figure 4 and Figure 6. Figure 4 displays the direct effects, and the model showed that older age and non-White race were negatively associated with motivation, while higher education showed a positive association. Figure 6 incorporated explanatory variables, revealing both direct and indirect pathways. This model demonstrated that health information distrust negatively influenced motivation, whereas having a regular healthcare provider had a positive effect.

The summary of the SEM results are presented in Table 5.

### 3.4. Results of SEMs’ Fit Statistics

The fit statistics of the SEMs showed that the inclusion of health system-related explanatory variables improved the model’s explanatory power. In the overall sample, the coefficient of determination increased from 0.050 in the model without explanatory variables to 0.285 in the model with explanatory variables (Appendix A Table A2). The fit statistics of the stratified SEMs indicated that the coefficient of determination increased from 0.055 in the model without explanatory variables to 0.286 in the model with explanatory variables. 

## 4. Discussion

In a nationally representative study of US adults, we found that certain health system factors, specifically, health information distrust, having a regular healthcare provider, and the frequency of healthcare visits, mediated the relationship between sociodemographic and health characteristics and the motivation to participate in clinical trials. The findings revealed notable gender differences in how health system-related factors influence their motivation for clinical trial participation, highlighting the complex and nuanced ways in which men and women engage with health systems.

### 4.1. Healthcare Relations Explanatory Variables

In our study, for the overall sample, health information distrust emerged as a significant factor influencing motivation. Participants with a higher level of distrust in health information showed lower motivation. This finding is consistent with the existing literature, highlighting the critical role of trust in health systems in shaping individuals’ willingness to participate in medical research [47,48]. On the other hand, we observed a positive association between having a regular healthcare provider and motivation. This may stem from the strong, trusting relationships between patients and providers, often developed through regular and consistent healthcare visits [49]. This can increase patients’ confidence in medical recommendations, including clinical trial participation.

### 4.2. Gender Differences

The stratified analysis by gender also revealed significant differences in the factors influencing the motivation for clinical trial participation. While having a regular provider was positively associated with motivation among men, this finding was not observed among women, thereby indicating that initiatives to enhance participation among men might benefit from focusing on strengthening patient–provider relationships. Despite having regular interactions with healthcare providers, women often face barriers that reduce their participation in clinical trials. These barriers include heightened stigma, and economic and caregiving responsibilities that deprioritize their own health [50,51]. Additionally, traditional gender roles and provider bias may lead to the lower prioritization of treatment for women [52]. Moreover, healthcare policies may not sufficiently address women’s specific needs, such as childcare during treatment, contributing to lower motivation and engagement in clinical trials [53].

From the stratified results, while health information distrust showed no association with motivation in clinical trial participation among men, this emerged as a significant factor for women, where women with higher distrust showed less motivation. Moreover, considering the evidence that trust plays a key role in influencing the willingness to participate in medical research [54,55,56], the identified gender differences in distrust in health information and motivation might explain the disparities in women’s representation within clinical trials. This unique finding suggests that strategies might need to be tailored differently for women to build trust in health information and medical systems, and to encourage them to participate in clinical trials. Our study also found that highly educated women showed higher motivation to participate in clinical trials. This is potentially due to higher health literacy. Women with higher education often possess greater health literacy, which deepens their understanding of the benefits of medical research in advancing medical knowledge and effectively improving health outcomes for all people [57]. Those with higher education may also be influenced by altruism. Social psychological theories suggest that those with a strong orientation toward contributing to broader societal benefits or helping others are often engaged in prosocial behaviors [58]. Thus, they are more likely to be motivated by the societal benefits that clinical trials can offer, even when the personal benefits are minimal or indirect [59]. No significant association was found between higher education and motivation for men, pointing to a considerable gender-specific difference in the predictors of motivation. One of the surprising findings of this study was the low motivation among older men. This result differs from the common perceptions that older adults, with their experience, wisdom, and often greater sense of altruism, would be more motivated to participate in activities that benefit society [60]. The lower motivation could be due to the unique challenges experienced by the older adults. Clinical trials are often held in locations that are not easily accessible [61,62]. In addition, lower levels of health literacy [63]) and concerns (i.e., fear of discrimination [61], and family and friends being skeptical or uninformed about clinical trials) could diminish their motivation for participation.

The comparison between genders in this study provides valuable insights to inform recruitment strategies for clinical trials. For trials aiming to achieve gender balance, understanding that factors such as age, non-White race, education, health information distrust, and having a regular healthcare provider affect men and women differently can help to tailor outreach efforts. These findings are also relevant for sex-specific clinical trials, such as those focused on breast cancer or ovarian cancer. Identifying the factors that drive or hinder women’s motivation to participate can help to design targeted strategies to enhance enrollment in these studies, ultimately improving the diversity and representation in clinical trials.

### 4.3. Implications for Policies and Programs

Since distrust in health information emerged as a crucial factor in lowering the motivation to participate in clinical trials, especially among women, policies to rebuild trust in healthcare information and systems are essential. Educational campaigns aimed at addressing historical mistrust, and thereby increasing the willingness to participate in clinical research, should be prioritized. Educational campaigns should be tailored to address the historical context of mistrust [20]. For example, for Black communities, acknowledging events like the Tuskegee Syphilis Study and highlighting the protections for participants in place, such as IRB oversight and informed consent processes, can play a crucial role in rebuilding trust. Overall, these campaigns should emphasize transparency in the clinical trial process, the ethical safeguards, the voluntary nature of trial participation and withdrawal, and successful outcomes from previous clinical trials with diverse participant populations. In addition, hearing from previous participants is crucial for developing ethically informed research and increasing enrollment [64]. Employing community engagement efforts and maintaining culturally sensitive communication to address health information distrust may prove especially effective among women [20]. Community engagement strategies, such as partnering with trusted local organizations, faith-based groups, and community health workers, are highly effective in disseminating information about clinical trials [20]. Hosting workshops and Q&A sessions in familiar community settings can further clarify the benefits and safety measures associated with clinical trial participation.

For men, recruitment efforts could focus more on the patient–provider relationship, which is more sustainable and builds on robust trust. Providing healthcare providers with adequate resources, encouraging them to discuss clinical trial opportunities with eligible participants, and highlighting the personal benefits of participation, such as access to novel treatments, could be an effective approach. One recent study also emphasized the importance of educating healthcare providers about the benefits of clinical trials [65]. Initiatives to address structural barriers that limit participation, such as limited access to healthcare and the frequency of visits, should also be implemented. Expanding healthcare access through supplemental programs or enrollment incentives could offer more opportunities for the underrepresented population to learn and participate in clinical trials. There is also a need to tailor outreach efforts to the specific needs of underrepresented populations co-designed by community-based organizations and research institutions.

The findings of this study have significant long-term implications for future research, policies, programs, and practices. These results may inspire researchers to design longitudinal studies that examine how societal changes, shifts in perceptions of the healthcare system, and evolving policies influence motivation over time. Healthcare providers can use these insights to strengthen patient–provider relationships, build trust, and effectively communicate about clinical trial benefits and opportunities. Additionally, the findings could help reform or guide the development of new policies focused on increasing diversity by addressing structural barriers. Policymakers may prioritize funding for community-driven engagement programs, promoting initiatives that rebuild trust in the healthcare system, and expanding healthcare access.

These findings can also be used to identify subgroups that maximize recruitment efforts and guide strategies to improve clinical trial participation without increasing the burden on healthcare systems. Rather than increasing healthcare visits, these insights can target subgroups already motivated to participate, such as those with strong patient–provider relationships. Enhancing satisfaction during routine healthcare visits with a regular provider can further foster trust and engagement.

### 4.4. Implications for Future Research

Longitudinal studies on building trust in health information and the overall health system, with its effect on the willingness and participation in clinical trials, as well as the role of healthcare providers in recruitment, could provide deeper insights. Future studies can also take a qualitative approach to understanding how local contexts, historical events of unethical medical experiments, and cultural beliefs shape perceptions of clinical research among diverse groups. In addition, future research should also evaluate the effectiveness of strategies or policies aimed at increasing trust and patient–provider relationships to enhance diversity in clinical trials. Research in behavioral economics and personality studies has highlighted gender differences in self-confidence and their potential implications. Women with lower self-confidence may take a more cautious approach to decision making, which can manifest as distrust in the health system or reluctance to participate in healthcare initiatives that are less familiar or unknown [66,67]. Additionally, societal stereotypes about confidence can shape individual behaviors and perceptions, potentially leading women to undervalue their ability to assess or trust medical systems [68]. Future research examining the interplay between self-confidence, trust in health systems, and motivation to participate in clinical trials could provide valuable insights.

While our study provided crucial information on the motivators of clinical trial participation, the findings should be interpreted with the consideration of potential changes over time or across different contexts. Variations in cultural or geographic settings, new policies aimed at increasing diversity in clinical trials, or differences among other ethnic minorities could influence the predictors or health system-related explanatory variables of motivation. Additionally, the motivation to participate in clinical trials may vary depending on the type of trial. Future research should account for these dynamics to have a more comprehensive understanding of the motivators.

### 4.5. Limitations and Strengths

This study has some limitations. Due to the nature of the cross-sectional design, we could not establish causality. In addition, the study has a small sample size within specific subgroups such as other race categories. Additionally, not testing for interactions between variables of interest, which could better reveal the complex non-linear relationships between those variables, further constrains the study’s findings. Another limitation is self-selection bias; individuals who chose to respond to the survey may have different characteristics or motivations compared to those who did not. In addition, the low response rates may affect the generalizability and validity of the findings. However, HINTS is the only study that has measured motivation for clinical trial participation on a national scale. Despite the low response rate and these limitations, this study used a nationally representative sample, which enhanced the generalizability of the findings and provided crucial information on the mediating role of health system-related factors in shaping the motivation for clinical trial participation. 

## 5. Conclusions

In a nationally representative survey of US adults, we identified potential mechanisms through which health system-related factors influence the motivation to participate in clinical trials, such as relationships and trust in the healthcare system. Individuals’ relationships and trust in the healthcare system helped to explain why men and women differ in their interests and willingness to participate in clinical trials. We found that health information distrust negatively influenced motivation among women, while having a regular healthcare provider had positive effects among men. The findings highlighted the need for policymakers, healthcare providers, and researchers to prioritize strategies that address structural barriers, build trust, improve access, and foster inclusive participation in clinical trials. Additionally, our findings underscore the importance of gender-specific approaches that address differing relationships with the healthcare system. These insights can guide the development of more effective strategies to ensure that clinical trials are more representative and inclusive.

## Figures and Tables

**Table 1:** Measures of health relations explanatory variables.

Variable and Prompt	Response Coding	Analytical Strategy
Health Information Distrust In general, how much would you trust information about cancer from each of the following?— (a) A doctor; (b) Family or friends; (c) Government Health Agencies; (d) Charitable organizations; (e) Religious organizations or leaders.	For each option: A lot—coded as 1 Some—coded as 2 A little—coded as 3 Not at all—coded as 4	Mean score of items calculated as a continuous measure A higher score indicates greater distrust of health information
Having a Regular Provider Not including psychiatrists and other mental health professionals, is there a particular doctor, nurse, or other health professional that you see most often?	Yes—coded as 1 No—coded as 0	Dichotomous
Frequency of Healthcare Visits In the past 12 months, not counting the times you went to an emergency room, how many times did you go to a doctor, nurse, or other health professional to get care for yourself?	None—coded as 0 1 time—coded as 1 2 times—coded as 2 3 times—coded as 3 4 times—coded as 4 5 times—coded as 5 10 or more times—coded as 6	Continuous
Confidence in Obtaining Health Information Overall, how confident are you that you could get advice or information about cancer if you needed it?	Completely confident—coded as 1 Very confident—coded as 2 Somewhat confident—coded as 3 A little confident—coded as 4 Not confident at all—coded as 5	Continuous
Quality of Care Overall, how would you rate the quality of health care you received in the past 12 months?	Excellent—coded as 1 Very good—coded as 2 Good—coded as 3 Fair—coded as 4 Poor—coded as 5	Continuous
Communication with Health Professionals How often did they do each of the following?— (a) Give you the chance to ask all of the health-related questions you had; (b) Give the attention you needed to your feelings and emotions; (c) Involve you in decisions about your health care as much as you wanted; (d) Make sure you understood the things you needed to do to take care of your health; (e) Explain things in a way you could understand; (f) Spend enough time with you; (g) Help you deal with feelings of uncertainty about your health or health care.	Always—coded as 1 Usually—coded as 2 Sometimes—coded as 3 Never—coded as 4	Continuous

**Table 2:** Descriptive statistics in the overall sample and by motivation status.

Variables	Low Motivation (Weighted *n* = 124,447,744)			High Motivation (Weighted *n* = 125,078,757)			Total (Weighted *n* = 253,815,197)		
	%	SE	95% CI	%	SE	95% CI	%	SE	95% CI
Gender									
Men	50.0	0.02	45.6–54.5	47.7	0.02	43.7–51.8	48.6	0.02	45.6–51.7
Women	50.0	0.02	45.5–54.4	52.3	0.02	48.2–56.3	51.4	0.02	48.3–54.4
Race									
White	74.2	0.02	70.5–77.5	77.7	0.01	74.7–80.4	75.7	0.01	73.5–77.8
Non-White	25.8	0.01	22.5–29.5	22.3	0.01	19.6–25.3	24.3	0.01	22.2–26.5
Ethnicity									
Non-Hispanic	82.9	0.01	80.2–85.3	83.3	0.02	79.5–86.5	83.1	0.01	81.0–85.0
Hispanic	17.1	0.01	14.7–19.8	16.7	0.02	13.5–20.5	16.9	0.01	15.0–19.0
Educational Attainment									
Less than High School	11.7	0.01	9.1–15.0	4.2	0.01	2.9–6.1	8.0	0.01	6.4–10.0
High School Graduate	21.0	0.01	18.4–23.8	23.8	0.02	20.3–27.6	22.5	0.01	20.3–24.9
Some College	39.8	0.02	36.0–43.7	38.7	0.02	34.8–42.8	39.2	0.01	36.5–42.0
College Graduate or More	27.5	0.01	24.9–30.2	33.3	0.02	30.0–36.9	30.3	0.01	28.0–32.6
General Health Condition									
Poor or Fair	13.2	0.01	11.0–15.9	14.8	0.01	12.1–18.0	0.14	0.01	12.4–16.0
Excellent, Very good, or Good	86.8	0.01	84.1–89.0	85.2	0.01	82.0–87.9	0.86	0.01	84.0–87.6
Having Regular Provider									
No	41.4	0.02	37.9–45.0	33.9	0.02	30.0–38.0	37.7	0.01	35.1–40.5
Yes	58.6	0.02	55.0–62.1	66.1	0.02	62.0–70.0	62.3	0.01	59.5–64.9
Frequency of Healthcare Visits (Past 12 months)									
None	20.8	0.02	17.7–24.3	13.0	0.01	10.4–16.0	16.8	0.01	14.8–19.1
1 time	15.1	0.01	12.4–18.3	18.7	0.02	15.3–22.5	16.8	0.01	14.4–19.4
2 times	21.1	0.02	18.0–24.5	19.5	0.01	17.2–22.1	20.2	0.01	18.2–22.4
3 times	12.1	0.01	10.2–14.3	14.8	0.01	12.3–17.7	13.4	0.01	11.8–15.1
4 times	10.7	0.01	9.1–12.6	11.2	0.01	09.2–13.7	11.1	0.01	9.7–12.6
5–9 times	13.0	0.01	10.6–15.9	14.3	0.01	12.0–17.0	13.7	0.01	12.0–15.7
10 or more times	7.2	0.01	5.7–9.1	8.5	0.01	6.7–10.7	8.0	0.01	6.7–9.4
Quality of Care									
Excellent	29.4	0.02	25.7–33.5	27.7	0.02	24.5–31.1	28.3	0.01	25.9–30.8
Very Good	40.8	0.02	37.3–44.5	42.5	0.02	38.4–46.8	41.9	0.01	39.1–44.7
Good	23.4	0.02	19.6–27.8	20.9	0.02	17.6–24.6	22.2	0.01	19.6–25.0
Fair	5.1	0.01	3.4–7.4	8.3	0.02	5.4–12.4	6.7	0.01	5.0–8.8
Poor	1.3	0.00	0.6–2.6	0.6	0.00	0.4–1.2	01.0	0.00	0.6–1.7
Confidence in Obtaining Health Information									
Completely Confident	30.8	0.02	27.3–34.6	31.0	0.02	27.6–34.5	30.9	0.01	28.5–33.3
Very Confident	32.0	0.01	29.3–34.8	38.4	0.02	34.7–42.3	35.1	0.01	32.9–37.3
Somewhat Confident	28.3	0.02	25.0–31.8	23.7	0.02	20.8–27.0	26.1	0.01	24.0–28.4
A Little Confident	5.5	0.01	04.2–07.2	4.5	0.01	2.9–7.1	5.0	0.01	3.9–6.4
Not Confident At All	3.4	0.01	1.9–5.7	2.4	0.01	1.2–4.5	2.9	0.01	1.9–4.4
	**Mean**	**SE**	**95% CI**	**Mean**	**SE**	**95% CI**	**Mean**	**SE**	**95% CI**
Age (years)	50.1	0.89	48.4–51.9	46.4	0.73	44.9–47.8	48.4	0.53	47.4–49.5
Depression	2.0	0.12	1.8–2.3	2.4	0.13	2.1–2.6	2.2	0.09	2.0–2.4
Health Information Distrust	2.2	0.02	2.2–2.3	2.2	0.02	2.1–2.2	2.2	0.02	2.2–2.3
Communication with Health Professionals (1–4)	1.6	0.03	1.6–1.7	1.6	0.03	1.6–1.7	1.6	0.02	1.6–1.7

Note. SE stands for standard error; CI stands for confidence interval.

**Table 3:** Summary of the results in the pooled sample without and with explanatory variables.

Variables	Model 1 Without Explanatory Variables		Model 2 With Explanatory Variables	
	β	SE	β	SE
Outcome: Motivation for Clinical Trial Participation				
Age	−0.144 ***	0.038	−0.170 ***	0.038
Gender (Women)	0.017	0.030	0.008	0.029
Race (Non-White)	−0.078 **	0.026	−0.078 **	0.027
Ethnicity (Hispanic)	−0.059	0.031	−0.051	0.032
Education	0.125 ***	0.029	0.117 ***	0.029
General Health Condition	−0.058 *	0.026	−0.053 *	0.026
Depression	0.042	0.031	0.046	0.031
Health Information Distrust	NA	NA	−0.094 **	0.028
Having a Regular Provider	NA	NA	0.087 **	0.028
Frequency of Healthcare Visits (past 12 months)	NA	NA	0.014	0.029
Outcome: Health Information Distrust				
Age	NA	NA	0.017	0.036
Gender (Women)	NA	NA	−0.073 *	0.030
Race (Non-White)	NA	NA	−0.060	0.034
Ethnicity (Hispanic)	NA	NA	−0.028	0.033
Education	NA	NA	0.010	0.036
General Health Condition	NA	NA	−0.004	0.031
Depression	NA	NA	0.084 **	0.032
Outcome: Having Regular Provider				
Age	NA	NA	0.286 ***	0.031
Gender (Women)	NA	NA	0.030	0.031
Race (Non-White)			−0.070	0.033
Ethnicity (Hispanic)	NA	NA	−0.121 *	0.035
Education	NA	NA	0.077 **	0.025
General Health Condition	NA	NA	−0.041	0.026
Depression	NA	NA	0.024	0.026
Outcome: Frequency of Healthcare Visits (Past 12 months)				
Age	NA	NA	0.217 ***	0.027
Gender (Women)	NA	NA	0.064 **	0.022
Race (Non-White)			−0.008	0.029
Ethnicity	NA	NA	−0.083 **	0.031
Education	NA	NA	0.128 ***	0.025
General Health Condition	NA	NA	−0.143 ***	0.030
Depression	NA	NA	0.161 ***	0.027

Note. SE stands for standard error; β stands for beta coefficient. *** *p* < 0.001; ** *p* < 0.01, and * *p* < 0.05.

**Table 4:** Summary of the group analysis results by the gender results without and with explanatory variables.

Variables	Men				Women			
	Model 3 Without Explanatory Variables		Model 4 With Explanatory Variables		Model 5 Without Explanatory Variables		Model 6 With Explanatory Variables	
	β	SE	β	SE	β	SE	β	SE
Outcome: Motivation for Clinical Trial Participation								
Age	−0.219 ***	0.046	−0.275 ***	0.045	−0.063	0.055	−0.076	0.052
Race (Non-White)	−0.073 *	0.036	−0.068	0.035	−0.085 *	0.037	−0.087 *	0.039
Ethnicity (Hispanic)	−0.099 *	0.042	−0.099 *	0.042	−0.011	0.044	0.007	0.045
Education	0.077	0.044	0.057	0.043	0.175 ***	0.042	0.178 ***	0.042
General Health Condition	−0.056	0.039	−0.051	0.039	−0.048	0.033	−0.038	0.033
Depression	0.064	0.041	0.052	0.042	0.021	0.042	0.034	0.040
Health Information Distrust	NA	NA	−0.047	0.042	NA	NA	−0.125 **	0.037
Having a Regular Provider	NA	NA	0.116 **	0.041	NA	NA	0.082	0.043
Frequency of Healthcare Visits (past 12 months)	NA	NA	0.043	0.039	NA	NA	−0.005	0.040
Outcome: Health Information Distrust								
Age	NA	NA	0.018	0.052	NA	NA	0.024	0.041
Race (Non-White)	NA	NA	−0.058	0.045	NA	NA	−0.070	0.044
Ethnicity (Hispanic)	NA	NA	−0.109 *	0.048	NA	NA	0.056	0.041
Education	NA	NA	−0.036	0.061	NA	NA	0.048	0.033
General Health Condition	NA	NA	−0.065	0.042	NA	NA	0.047	0.044
Depression	NA	NA	0.049	0.048	NA	NA	0.107 **	0.039
Outcome: Having Regular Provider								
Age	NA	NA	0.350 ***	0.041	NA	NA	0.213 ***	0.043
Race (Non-White)	NA	NA	−0.071	0.054	NA	NA	−0.058	0.035
Ethnicity (Hispanic)	NA	NA	−0.056	0.057	NA	NA	−0.187 ***	0.043
Education	NA	NA	0.108 **	0.040	NA	NA	0.042	0.033
General Health Condition	NA	NA	−0.029	0.047	NA	NA	−0.059	0.033
Depression	NA	NA	0.015	0.047	NA	NA	0.031	0.034
Outcome: Frequency of Healthcare Visits (Past 12 months)								
Age	NA	NA	0.306 ***	0.038	NA	NA	0.139 **	0.040
Race (Non-White)	NA	NA	−0.041	0.042	NA	NA	0.013	0.038
Ethnicity (Hispanic)	NA	NA	−0.054	0.041	NA	NA	−0.103 *	0.042
Education	NA	NA	0.138 ***	0.035	NA	NA	0.104 **	0.035
General Health Condition	NA	NA	−0.165 **	0.051	NA	NA	−0.134 **	0.038
Depression	NA	NA	0.223 ***	0.047	NA	NA	0.110 **	0.031

Note. SE stands for standard error; β stands for beta coefficient. *** *p* < 0.001; ** *p* < 0.01, and * *p* < 0.05.

**Table 5:** Summary of the SEM results in the overall sample and across groups.

Variables	Overall Sample		Men		Women	
	Without Explanatory Variables	With Explanatory Variables	Without Explanatory Variables	With Explanatory Variables	Without Explanatory Variables	With Explanatory Variables
Age	-	-	-	-	NS	NS
Non-White Race	-	-	-	NS	-	-
Education	+	+	NS	NS	+	+
Health Information Distrust	NA	-	NA	NS	NA	-
Having a Regular Healthcare Provider	NA	+	NA	+	NA	NS
Frequency of Healthcare Visits	NA	NS	NA	NS	NA	NS

Note: “-” is a negative association; “+” is a positive association; NS stands for not significant; NA stands for not applicable.

## Data Availability

HINTS is publicly available at https://hints.cancer.gov/ (accessed on 27 February 2024).

## Appendix A

**Table A1:** Summary of the initial SEM results with all of the explanatory variables.

Variables	Overall Sample	Stratified Sample				
		Men			Women	
	β	SE	β	SE	β	SE
Outcome: Motivation for Clinical Trial Participation						
Age	−0.167 ***	0.037	−0.271 ***	0.044	−0.075	0.052
Gender (Women)	0.008	0.029	NA	NA	NA	NA
Race (Non-White)	−0.079 **	0.027	−0.069	0.035	−0.088 *	0.038
Ethnicity (Hispanic)	−0.051	0.031	−0.101 *	0.043	0.010	0.045
Education	0.120	0.030	0.060	0.043	0.181 ***	0.043
General Health Condition	−0.052 *	0.026	−0.045	0.040	−0.038	0.033
Depression	0.053	0.031	0.052	0.043	0.043	0.039
Health Information Distrust	−0.085 **	0.028	−0.043	0.043	−0.115 **	0.034
Having a Regular Provider	0.085 **	0.028	0.117 **	0.041	0.081	0.043
Frequency of Healthcare Visits (past 12 months)	0.016	0.028	0.046	0.038	−0.005	0.040
Quality of Care	0.029	0.040	0.050	0.053	0.021	0.048
Confidence in Obtaining Health Information	−0.022	0.031	−0.018	0.047	−0.037	0.040
Communication with Health Professionals	−0.050	0.033	−0.038	0.050	−0.048	0.054
Outcome: Health Information Distrust						
Age	0.017	0.036	0.018	0.052	0.024	0.041
Gender (Women)	−0.073 *	0.030	NA	NA	NA	NA
Race (Non-White)	−0.060	0.034	−0.058	0.045	−0.070	0.044
Ethnicity (Hispanic)	−0.028	0.033	−0.109 *	0.048	0.056	0.041
Education	0.010	0.036	−0.036	0.061	0.048	0.033
General Health Condition	−0.004	0.031	−0.065	0.042	0.047	0.044
Depression	0.084 **	0.032	0.049	0.048	0.107 **	0.039
Outcome: Having Regular Provider						
Age	0.286 ***	0.031	0.350 ***	0.041	0.213 ***	0.043
Gender (Women)	0.030	0.031	NA	NA	NA	NA
Race (Non-White)	−0.070	0.033	−0.071	0.054	−0.058	0.035
Ethnicity (Hispanic)	−0.121 *	0.035	−0.056	0.057	−0.187 ***	0.043
Education	0.077 **	0.025	0.108 **	0.040	0.042	0.033
General Health Condition	−0.041	0.026	−0.029	0.047	−0.059	0.033
Depression	0.024	0.026	0.015	0.047	0.031	0.034
Outcome: Frequency of Healthcare Visits (Past 12 months)						
Age	0.217 ***	0.027	0.306 ***	0.038	0.139 **	0.040
Gender (Women)	0.064 **	0.022	NA	NA	NA	NA
Race (Non-White)	−0.008	0.029	−0.041	0.042	0.013	0.038
Ethnicity	−0.083 **	0.031	−0.054	0.041	−0.103 *	0.042
Education	0.128 ***	0.025	0.138 ***	0.035	0.104 **	0.035
General Health Condition	−0.143 ***	0.030	−0.165 **	0.051	−0.134 **	0.038
Depression	0.161 ***	0.027	0.223 ***	0.047	0.110 **	0.031
Quality of Care						
Age	−0.100 **	0.034	−0.102	0.064	−0.083	0.043
Gender (Women)	−0.055	0.031	NA	NA	NA	NA
Race (Non-White)	0.039	0.034	0.019	0.058	0.052	0.039
Ethnicity	0.091 **	0.032	0.106	0.055	0.089 *	0.039
Education	−0.066	0.036	−0.067	0.056	−0.060	0.040
General Health Condition	−0.152 ***	0.032	−0.171 **	0.059	−0.148 ***	0.041
Depression	0.120 **	0.036	0.196 **	0.064	0.062	0.035
Confidence in Obtaining Health Information						
Age	0.018	0.037	−0.011	0.054	0.063	0.039
Gender (Women)	−0.059	0.031	NA	NA	NA	NA
Race (Non-White)	0.015	0.031	−0.000	0.051	0.025	0.034
Ethnicity	0.032	0.039	0.011	0.065	0.047	0.041
Education	−0.091 *	0.039	−0.134 *	0.058	−0.051	0.035
General Health Condition	−0.004	0.022	−0.013	0.040	0.020	0.031
Depression	0.151 ***	0.030	0.158 ***	0.044	0.154 ***	0.037
Communication with Health Professionals						
Age	−0.030	0.041	−0.001	0.067	−0.049	0.045
Gender (Women)	−0.028	0.032	NA	NA	NA	NA
Race (Non-White)	0.003	0.033	0.010	0.053	−0.010	0.037
Ethnicity	0.065	0.038	0.059	0.069	0.080	0.041
Education	0.046	0.036	0.041	0.058	0.046	0.044
General Health Condition	−0.068 *	0.028	−0.069	0.047	−0.077	0.040
Depression	0.169 ***	0.031	0.221 ***	0.049	0.117 **	0.038

Note. SE stands for standard error; β stands for beta coefficient. *** *p* < 0.001; ** *p* < 0.01, and * *p* < 0.05.

**Table A2:** The structural equation models’ fit statistics.

Models	Coefficient of Determination
Overall Sample	
SEM in the Overall Sample without Explanatory Variables	0.050
SEM in the Overall Sample with Explanatory Variables	0.285
SEM by Gender	
SEM by Gender without Explanatory Variables	0.055
SEM by Gender with Explanatory Variables	0.286
